# Global Linearized Sparse Prediction and Adaptive Dead Zone Compensation for a Piezoelectric Actuator

**DOI:** 10.3390/mi17040392

**Published:** 2026-03-24

**Authors:** Xue Qi, Meiting Zhao, Lina Zhang, Lei Fan, Zhihui Liu, Pengying Xu, Qiulin Tan

**Affiliations:** 1 Key Laboratory of Micro/Nano Devices and Systems, Ministry of Education, North University of China, Taiyuan 030051, China; 13068079915@163.com (M.Z.); lnzhang0810@163.com (L.Z.); 20230054@nuc.edu.cn (L.F.); 20250183@nuc.edu.cn (Z.L.); tanqiulin@nuc.edu.cn (Q.T.); 2 State Key Laboratory of Extreme Environment Optoelectronic Dynamic Measurement Technology and Instrument, North University of China, Taiyuan 030051, China; 3 Science and Technology on Electronic Test and Measurement Laboratory, North University of China, Taiyuan 030051, China

**Keywords:** global linearized sparse prediction, model predictive control, piezoelectric actuator, adaptive Kalman observer, dead zone compensation

## Abstract

A piezoelectric actuator (PEA) is a fundamental part of a high-precision motion system, yet its performance is critically constrained by inherent nonlinearities such as the velocity dead zone and hysteresis. To overcome these limitations and the associated time-varying dynamics, this study introduces a novel control framework for a dual-mode standing wave PEA. The framework integrates a Global Linearized Sparse Prediction (GLSP) model with an Adaptive Kalman Observer-based Model Predictive Control (AKOBMPC) strategy, specifically designed for velocity dead-zone compensation. The GLSP model employs Koopman operator theory to lift the complex, nonlinear electromechanical and contact dynamics into a linear invariant subspace. Incorporated with a deep learning-based structured pruning mechanism, the model achieves an effective balance between prediction accuracy and computational efficiency, facilitating real-time implementation. Leveraging this high-fidelity model, the AKOBMPC algorithm is developed to estimate unmeasurable disturbances and optimize the control sequence for precise velocity tracking. Experimental results demonstrate the GLSP model’s accurate prediction of system behavior under varying loads and excitation frequencies. The proposed controller effectively suppresses the velocity dead zone, achieving tracking errors within ±0.35 mm/s for a 40.00 mm/s trapezoidal reference and within ±0.50 mm/s for sinusoidal tracking. These results confirm the superior performance of the AKOBMPC scheme over conventional methods, offering a robust solution for high-precision velocity regulation in PEA system and contributing to the advancement of next-generation precision actuator.

## 1. Introduction

A piezoelectric actuator (PEA) serves as a fundamental component in a precision motion system and is critical to the operation of modern high-end equipment, such as ultra-precision manufacturing platforms, biomedical micromanipulation robots, and adaptive optical alignment systems [1,2]. Operating on the inverse piezoelectric effect, PEA directly transduces electrical energy into micro-nano scale mechanical displacement. It exhibits notable characteristics including fast response, high positioning accuracy, large output force, compact structure, and strong resistance to electromagnetic interference [3]. These attributes make it indispensable in applications demanding high precision, velocity, and stability, such as microelectronic packaging, scanning probe microscopy, and satellite mirror control [4,5,6,7]. With continuous advances in the performance of high-end equipment, especially in emerging fields like cross-scale manipulation, brain-computer interfaces, and in-situ metrology, the requirements for a PEA motion system in terms of higher precision, broader bandwidth, and higher stability are growing steadily [8,9,10,11,12].

However, the performance of a PEA is limited by inherent nonlinear characteristics, especially the velocity dead zone, which refers to the near-zero displacement output when the input voltage is below a specific threshold. The formation of the dead zone is fundamentally driven by the intrinsic hysteresis and creep of piezoelectric material. Hysteresis creates a phase lag between the input voltage and the strain output, while creep leads to a time-dependent drift in strain, both of which directly lead to dynamic shifts in the dead zone voltage threshold. This threshold varies dynamically with operating frequency, external load, and temperature, resulting in strongly coupled and time-varying nonlinear behavior [13,14,15]. Consequently, the dead zone not only introduces steady-state error and degrades low-velocity tracking accuracy, but may also excite oscillations or even lead to instability in closed-loop systems [16,17,18,19]. The system dynamics are further complicated by interactions with other nonlinearities like hysteresis, making the compensation of the dead zone a critical challenge in PEA control.

Significant studies have focused on modeling and mitigating the dead zone effects, primarily through high-fidelity modeling and advanced control strategies. From a modeling perspective, the integration of the dead zone operator into hysteresis frameworks, such as the asymmetric Bouc–Wen model, allows for a more accurate description of coupled dead zone phenomena [20,21]. Recent work has developed an improved Prandtl–Ishlinskii (PI) model for precision drive systems, which has a single-sided dead zone operator and provides a valuable reference for nonlinear modeling of a dead zone [22]. Further studies have explored rate-dependent hysteresis modeling and adaptive methods for selecting dead zone thresholds [23,24]. A modified PI model with a dead zone play operator and a Takagi–Sugeno fuzzy-based hybrid model have also been developed to enhance identifiability [25,26]. However, a fundamental constraint of these model-based schemes is the general absence of a tractable analytical inversion method for dynamic, rate-dependent PI models. As noted in [27], accurate inverse models are often unattainable, which severely restricts their practicality in high-speed dynamic control of PEA. For example, a compensatory Hammerstein structure enhanced by neural networks was introduced in [28], and a fractional Hammerstein model was proposed in [29] for rate-dependent nonlinear modeling in nano-manipulation, achieving high accuracy for complex hysteresis. However, these existing data-driven models are mostly black-box structures without a global linearized framework, which cannot balance high-precision dead zone and hysteresis prediction with low computational complexity for embedded real-time control of PEA system. Furthermore, linearization of the model is essential to facilitate the design of control strategy. The Koopman operator-based linearization techniques can represent complex nonlinear systems using deep learning, thus emerging as a promising method for modeling the characteristics of PEA [30].

In terms of control, both model-based and data-driven strategies have been investigated. To enhance tracking accuracy, a sliding mode controller with prescribed performance functions and an inverse dead zone compensator have been developed [31,32]. Adaptive control schemes have also been successfully applied to the PEA systems [33,34]. Alternative approaches include regulating velocity by adjusting the vibration trajectory of the driving foot [35], and implementing a robust anti-windup tracking controller on a custom nanostage for precision control [36]. Notably, intelligent control methods have shown effectiveness in dealing with dead zone uncertainties without the need for accurate models. For instance, robust trajectory tracking for composite standing wave PEA has been achieved by leveraging the complementary strengths of fuzzy logic and neural networks [37]. Another study combined an optimal PID control algorithm with an intelligent strategy [38]. Recently, the data-driven online learning-based Model Predictive Control (MPC) frameworks have emerged as a promising direction [39,40], including an MPC method developed specifically for piezoelectric motion stages using a Long Short-Term Memory (LSTM) model [41]. However, a common limitation of existing observer-based MPC schemes is their restricted ability to estimate and compensate for unmeasured, time-varying disturbances during operation. This shortcoming hinders the achievement of stable, high-precision speed tracking under complex and dynamic working conditions.

Despite these achievements, there are still some key issues. Most existing models consider dead zones as individual units rather than dynamic coupling phenomena. In addition, nonlinear ensemble compensation methods based on data-driven models have not been fully explored. Obviously, a clear data-driven framework is needed to combine complex modeling and control to achieve stable performance of PEA.

This study proposes a comprehensive framework for dead zone compensation in a PEA. The framework begins with an analysis of the underlying mechanisms and nonlinear characteristics of the dead zone. Subsequently, a global linearized modeling framework is established using the Koopman operator. Deep learning techniques are then integrated to identify model parameters in a data-driven manner, which facilitates the design of a high-performance closed-loop control algorithm. Experimental results demonstrate that the proposed method effectively suppresses velocity dead-zone effects, mitigates inherent nonlinearities, and significantly improves overall control accuracy.

The main innovations of this study are summarized as follows. First, a Koopman operator-based global linearization framework is developed to embed the nonlinear dynamics of the PEA into a linear invariant subspace. This approach addresses the poor working-condition adaptability of traditional dead-zone inverse models while enhancing the physical interpretability often lacking in purely data-driven methods. The resulting model maintains a Root Mean Square Velocity Modeling Error (RMSVME) below 0.32 mm/s under loads ranging from 0 to 2.0 N. Second, a structured pruning strategy is implemented within the proposed Global Linearized Sparse Prediction (GLSP) model, effectively balancing prediction fidelity with computational efficiency to enable real-time embedded applications. Finally, a MPC scheme integrated with an Adaptive Kalman Observer (AKOB) is proposed for the compensation of real-time, time-varying disturbances. This design overcomes the inherent steady-state error limitations associated with conventional observer-based MPC and feedforward compensation schemes. For a 100.00 mm/s sinusoidal reference, the controller achieves a maximum tracking error of 0.50 mm/s and a Steady-State Root Mean Square Velocity Error (SRMSVE) of 0.11 mm/s, representing a reduction in maximum tracking error of more than 44% compared to previous control methods.

## 2. Materials and Methods

**Figure 1:**
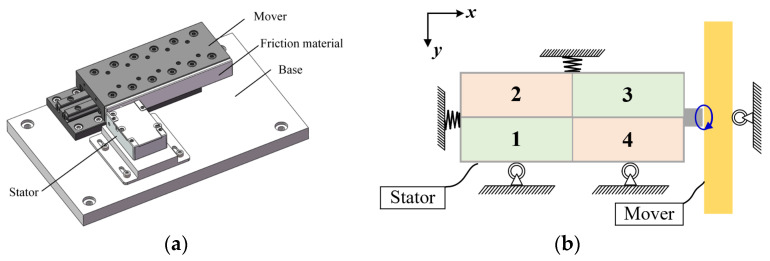
(**a**) The structure of PEA system; (**b**) Schematic of the system.

Figure 1a illustrates the structure of the bimodal standing wave PEA (Nanomotion Ltd., Yokneam Illit, Israel, Nanomotion, SE4 series) investigated in this study, which operates by exciting the first-order longitudinal and the second-order bending vibration modes. The system consists of the stator and the mover coated with friction material. As depicted in Figure 1b, under the action of preload, the elliptical motion generated by the driving foot is converted into linear motion of the rotor through frictional coupling.

### 2.1. Electromechanical Coupling Model of Stator

The overall model can be divided into the electromechanical coupling model of the stator, the contact interaction model, and the kinematic model of the mover. The dynamic behavior of the stator is formulated as

(1) $$M\overset{¨}{q}+C\overset{˙}{q}+Kq=\Theta \mu +F,$$

where ***M***, ***C***, and ***K*** denote the mass, structural damping, and stiffness matrices, respectively. The modal amplitude vector and the external excitation voltage vector are defined as ***q*** = [*q*_1_, *q*_2_]^T^ and ***u*** = [*u*_A_, *u*_B_]^T^, respectively. **Θ** = [*Θ*_L1_, *Θ*_L2_; *Θ*_B1_, *Θ*_B2_] represents the electromechanical coupling coefficient matrix, in which the subscripts *L* and *B* refer to the longitudinal and bending modes, respectively. ***F*** is the modal force vector associated with the axial force *F*_N_ and lateral force *F*_T_ at the contact interface.

The two-phase equivalent capacitance matrix is given by ***C***_p_ = diag(***C***_pA_, ***C***_pB_) and the charge vector ***Q*** = [*Q*_A_,*Q*_B_]^T^. The two-phase currents *i*_A_ and *i*_B_ satisfy

(2) $$i={\left[i_{A},i_{B}\right]}^{T}=\dot{Q}=\Theta^{T}\dot{q}+C_{p}\dot{u}$$

The contact model of the system is

(3) $$m\dot{v}+cv=F_{T}-F_{L}$$

where *m* and *c* are the mass and viscous damping coefficient of the mover, respectively. *v* stands for the output velocity of the mover, and *F*_L_ is the load force.

Owing to the intermittent nature of the contact model, *F*_T_ in Equation (3) constitutes a highly nonlinear term that is difficult to accurately describe.

### 2.2. Linearized Modeling

The PEA operates based on the inverse piezoelectric effect of piezoelectric materials, where the applied voltage induces strain to drive mechanical motion. This process involves strong electromechanical coupling between the electric field, structural dynamics and contact mechanics, which is the core source of inherent nonlinearity and time-varying disturbances in the system. The inherent hysteresis and creep of piezoelectric materials further exacerbate the asymmetric dead zone effect, leading to dynamic shifts in the dead zone voltage threshold under varying operating conditions, which are fully considered in the subsequent global linearized modeling.

**Figure 2:**
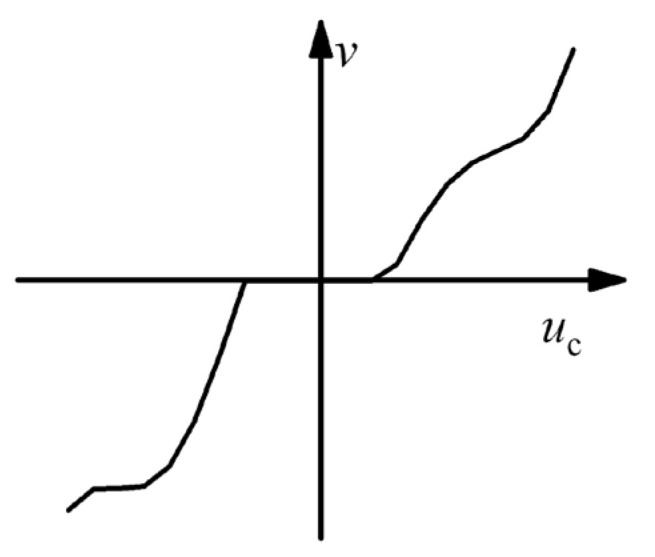
Schematic diagram of velocity regulation dead zone.

The nonlinear contact characteristics of the PEA system manifest as a regulation dead zone and pronounced nonlinearity in the output velocity. This not only degrades the system’s control accuracy but also may induce structural instability. As illustrated in Figure 2, the asymmetric nonlinear dead zone can be described as

(4) $$v=\left\{\begin{array}{ll} g_{l}\left(u_{c}\right), & u_{c}<d_{l}\\ 0, & d_{u}\leq u_{c}\leq d_{l}\\ g_{u}\left(u_{c}\right), & u_{c}>d_{u} \end{array}\right.,$$

where *u*_c_ denotes the control voltage. *d*_l_ and *d*_u_ represent the lower and upper voltage thresholds of the dead zone, respectively. The functions *g*_l_ and *g*_u_ are general nonlinear mapping symbols, which characterize the input–output relationship between the control voltage and the output velocity, respectively. Affected by the coupling of multi-physical factors including contact hysteresis, assembly errors, and mechanical friction, this nonlinear mapping has no deterministic explicit analytical expression. Therefore, this study focuses on the high-precision modeling of these complex velocity dead zone characteristics.

Global linearized Koopman theory is adopted to linearize the nonlinear output velocity characteristic of the system. According to Equations (1)–(3), the velocity *v* is correlated with the driving force and load force, while the driving force is dependent on the time derivatives of the stator’s modal coordinates. Consequently, the nonlinear mapping between the control voltage and the velocity can be discretized at the *k*-th time step as

(5) $$x_{k}=f\left(x_{k-1},u_{k-1}\right),$$

where the state variable is $x={\left[\nu ,i_{A},i_{B},F_{L}\right]}^{T}$, and the control variable is $u={\left[u_{AC},u_{Bc}\right]}^{T}$.

Based on Koopman theory, there exists an observable function that embeds the original nonlinear dynamics into a linear system in an infinite dimensional Hilbert space. The input vector is defined as **ϒ** = [***x***^T^, ***u***^T^]^T^, and the system dynamics can be represented by the linear evolution of the Koopman operator as

(6) $$g\left({ϒ}_{k}\right)=K\cdot g\left({ϒ}_{k-1}\right),$$

where *g*(**ϒ**) = [*g*_l_,…,*g_n_*_z+*n*u_]^T^ = [***ψ***(***x***)^T^, ***u***^T^]^T^ represents the lifting state vector and ***ψ*** = [***ψ***_1_,…, ***ψ****_n_*_z_]^T^ represents the set of *n*_z_-dimensional lifting function. The linear evolution in the invariant subspace is then expressed as

(7) $$z_{k}=\left[\begin{array}{c} \psi \left(x_{k}\right)\\ u_{k} \end{array}\right]=K\cdot \left[\begin{array}{c} \psi \left(x_{k-1}\right)\\ u_{k-1} \end{array}\right],$$

The resulting linear state-space representation is defined as

(8) $$z_{k}=M_{K}z_{k-1}+N_{K}u_{k-1},$$

where $M_{K}\in {\mathbb{R}}^{n_{z}\times n_{z}}$ and $N_{K}\in {\mathbb{R}}^{n_{z}\times n_{u}}$ denote the state transition matrix and the control parameter matrix, respectively.

The output variable is defined as

(9) $$y_{k}=C_{y}z_{k}\in {\mathbb{R}}^{n_{y}},$$

where the output matrix ***C***_y_ = [**1**, **0**_1×(*n*z-1)_] is designed to extract the velocity component from the lifting vector. The next objective is to identify the lifting function and the parameter matrices of the global linearized predictor for output velocity.

### 2.3. GLSP Model

To reduce the inherent bias in the selection of observation functions, a deep learning-based recognition framework is adopted. Through the general approximation ability of neural network, the Koopman operator conducts recognition in a data-driven way. This model embeds the dead zone nonlinear dynamics caused by hysteresis and creep into a linear invariant subspace through Koopman enhancement, achieving high fidelity characterization of material related inherent nonlinearity without the need for complex analytical models. According to Equation (5), the dimension of the input state vector is relatively high. The corresponding increase in computational complexity is not conducive to designing and implementing real-time controller. To balance prediction accuracy with hardware efficiency, a Neural Network-based GLSP model is proposed.

**Figure 3:**
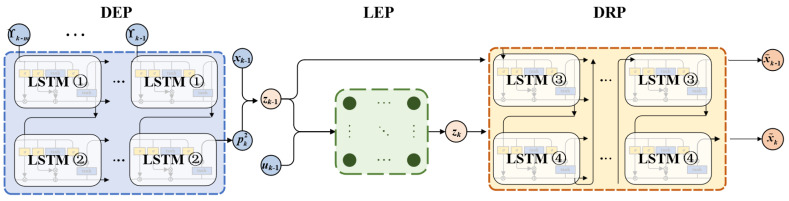
Diagram of the GLSP model.

As shown in Figure 3, the GLSP model is a variation of the autoencoder architecture, which is composed of the Dimension Enhancement Part (DEP), the Linear Evolution Part (LEP), and the Dimension Restoration Part (DRP). Both DEP and DRP utilize a two-layer LSTM structure, refers to LSTM ①–④ units.

DEP is acted as a nonlinear mapping to embed the original nonlinear state into a linear invariant subspace. LEP is to achieve linear evolution in invariant subspace. The lifting state vector and the prediction state vector are defined as

(10) $$z_{k-1}={\left[x_{k-1}^{T},{\left(p_{k-1}^{2}\right)}^{T}\right]}^{T},p_{k-1}^{2}=\Lambda ({ϒ}_{k-1},\ldots ,{ϒ}_{k-m}),$$

(11) $$z_{k}=K\cdot {\left[z_{k-1}^{T},u_{k-1}^{T}\right]}^{T}=[M_{K},N_{K}]\cdot {\left[z_{k-1}^{T},u_{k-1}^{T}\right]}^{T},$$

where $p_{k-1}^{2}$ is the output of the two-layer LSTM unit.

DRP is applied to ensure that the observation state variables of the invariant subspace retain most of the useful information of the original nonlinear system. The prediction output and reconstruction output are given by

(12) $${\tilde{x}}_{k}=\tilde{\Lambda }\left(z_{k}\right)=p_{k}^{4},$$

(13) $${\tilde{x}}_{k-1}=\tilde{\Lambda }\left(z_{k-1}\right)=p_{k-1}^{4},$$

As an evaluation metric for training, the cost function consists of reconstruction loss, linear loss, and prediction loss:

(14) $$L_{\mathrm{total}}=s_{1}L_{\mathrm{rec}}+s_{2}L_{\mathrm{lin}}+s_{3}L_{\mathrm{pred}},$$

(15) $$L_{\mathrm{rec}}={\left\|x_{k-1}-\tilde{\Lambda }\left(z_{k-1}\right)\right\|}_{2}^{2},$$

(16) $$L_{\mathrm{lin}}=\sum_{j=0}^{N-1}\frac{1}{N}{\left\|z_{k+j}-M_{K}^{j+1}z_{k-1}-N_{K}^{j+1}u_{k-1}\right\|}_{2}^{2},$$

(17) $$L_{\mathrm{pred}}=\sum_{j=0}^{N-1}\frac{1}{N}{\left\|x_{k+j}-\tilde{\Lambda }\left(M_{K}^{j+1}z_{k-1}+N_{K}^{j+1}u_{k-1}\right)\right\|}_{2}^{2},$$

where *s*_1_, *s*_2_ and *s*_3_ are the weight coefficients optimized for different losses.

The computational complexity and memory of the unit dominate the entire model. Table 1 lists the computational complexity, for an input dimension *n* and the number of hidden layer neural nodes in the two-layer LSTM structure is *h*_1_ and *h*_2_, the total cache is 4((*n* + *h*_1_)·*h*_1_+ (*h*_1_ + *h*_2_)·*h*_2_)+ 8(*h*_1_ + *h*_2_).

A structured pruning algorithm is introduced to optimize algorithmic efficiency and prevent overfitting. Algorithm 1 shows the pruning training algorithm. The sparsity is

(18) $$S_{t}=S_{f}+\left(S_{0}-S_{f}\right)\cdot {\left(1-\frac{\left(t-t_{0}\right)}{n\cdot \Delta t}\right)}^{3},$$

where *S*_0_ and *S*_1_ are the initial sparsity and final sparsity. *n* is the total number of pruning steps, which depends on the learning rate curve, *t* is the training steps. *t*_0_ is the initial step of the sparsity function, determined by the number of steps of the initial training benchmark network. Δ*t* is the pruning frequency, which is the frequency of updating the mask matrix, is counted in global steps.
**Algorithm 1.** Pruning Training AlgorithmInitialization:Initial the initial pruning rate *S*_0_ and the update frequency Δ*t*, *t* = 0;Step 1The initial network model is obtained through data-based training. Let *t*_0_ denotes the total number of steps required to obtain the converged benchmark model;Step 2Introduce a binary mask matrix to the LSTM layers of the network to facilitate weight pruning;Step 3Prune each block in the matrix and induce the weight matrix to independently prune according to each threshold, ensuring that each block has the same sparsity;Step 4Retrain the sparse network to recover model accuracy. Then adjust the remaining sparse weight parameters, and maintain the pruning weight parameters;Step 5Increase the pruning rate and return to Step 3 until the sparsity reaches the preset value, and the model accuracy is optimized.

## 3. Dead Zone Compensation Control

The Adaptive Kalman Observer-based Model Predictive Control (AKOBMPC) algorithm is proposed based on the GLSP model. The algorithm mitigates the residual interference of piezoelectric hysteresis and creep on dead zone compensation accuracy, which is achieved through AKOB-based real-time disturbance estimation combined with MPC-based rolling optimization of the control sequence. The AKOB in Algorithm 2 is implemented to detect the disturbance of the velocity *v* and two-phase current ***i***. The estimation of prior state ${\tilde{z}}_{k}$ and the output vector ${\tilde{y}}_{k}$ are

(19) $$\left\{\begin{array}{l} {\tilde{z}}_{k}=M_{K}{\hat{z}}_{k-1}+N_{K}u_{k-1}\\ {\tilde{y}}_{k}=C_{K}{\tilde{z}}_{k} \end{array}\right.,$$

It is assumed that the prediction noise and the measurement noise are both zero-mean white noise and are uncorrelated. As described in Algorithm 2, $\lambda_{k}=\frac{1-\zeta }{1-\zeta^{k+1}}$ is to update the noise covariance matrices ***Q*** and ***R***, *ζ* is the hyperparameter forgetting factor, which is [0.95, 0.99].
**Algorithm 2.** AKOB Algorithm**Definition****Parameter quantity**Initialize:Set the initial values of ***x***_0_, ***u***_0_, Set the initial values of the forgetting factor *λ*_0_, ***P***_0_, ***Q***_0_ and ***R***_0_
for *k* = 0:*N* begin
1: *k* = *k* + 1, the output of the GLSP model is set as the state estimation value ${\hat{z}}_{k-1}$;
2: calculate ${\tilde{z}}_{k}$ and ${\tilde{y}}_{k}$ based on Equation (19);
3: calculate the output error and the prior estimation covariance estimation $\delta_{k}={\tilde{y}}_{k}-C_{K}{\tilde{z}}_{k}$, ${\tilde{P}}_{k}=\mathrm{cov}\left(\delta_{k}\right)=E\left(\delta_{k}\delta_{k}^{T}\right)=M_{K}P_{k-1}M_{K}^{T}+Q_{k-1}$;
4: calculate the Kalman gain matrix $L_{k}={\tilde{P}}_{k}C_{K}^{T}{\left(C_{K}P_{k-1}C_{K}^{T}+R_{k}\right)}^{-1}$;
5: calculate the correction of ${\hat{z}}_{k}={\tilde{z}}_{k}+L_{k}\delta_{k}$;
6: calculate the residual $\varepsilon_{k}={\tilde{y}}_{k}-C_{K}{\hat{z}}_{k}$;
7: update the posterior estimate covariance matrix
$P_{k}=\mathrm{cov}\left(\varepsilon_{k}\right)=E\left(\varepsilon_{k}\varepsilon_{k}^{T}\right)=\left(I-L_{k}C_{K}\right){\tilde{P}}_{k}$;
8: update the adaptive estimation of covariance 
$R_{k}=\left(1-\lambda_{k}\right)R_{k-1}+\lambda_{k}\left(\varepsilon_{k}\varepsilon_{k}^{T}+C_{K}{\tilde{P}}_{k}C_{K}\right)$, $Q_{k}=\left(1-\lambda_{k}\right)Q_{k-1}+\lambda_{k}L_{k}\delta_{k}\delta_{k}^{T}L_{k}^{T}$.

The prediction horizon and the control horizon of the MPC method are set to *M* and *M*_u_, respectively. According to the optimal control law, the objective function is

(20) $$J={\left(\tilde{y}-y_{\mathrm{rd}}\right)}^{T}S_{p}p(\tilde{y}-y_{\mathrm{rd}})+\Delta u^{T}S_{q}\Delta u,$$

where the reference trajectory $y_{\mathrm{rd}}={\left[\begin{array}{cccc} y_{rd\left(k+1\right)}^{T} & y_{rd\left(k+2\right)}^{T} & \ldots & y_{rd\left(k+M\right)}^{T} \end{array}\right]}^{T}$. $\tilde{y}$ is the corrected output prediction, defined as ${\tilde{y}}_{k}=C_{y}{\tilde{z}}_{k}$, where ${\tilde{z}}_{k}$ is the correction of prior state estimation value. The weight coefficients for output error and control increment can be expressed as $S_{p}=diag\left(S_{p1},\ldots ,S_{\mathrm{pM}}\right)$ and $S_{q}=diag\left(S_{q1},\ldots ,S_{qM_{u}}\right)$.

Take the derivative of the objective function, and set ∂J/∂Δu=0 Retain the terms relevant to the optimization goal, and a step-by-step control strategy is employed to obtain the optimal control sequence. To enhance online computational efficiency, the control increment is determined as

(21) $$\Delta u_{k+i}=\alpha_{s}^{i}\Delta u_{k},i=1,\ldots ,M_{u}-1,$$

where *α*_s_ is the step factor that balances system robustness and response velocity.

The control voltage incremental sequence in the control horizon is

(22) $$\Delta u=\alpha_{s}\Delta u_{k}={\left[\begin{array}{cccc} 1 & \alpha_{s} & \ldots & \alpha_{s}^{M_{u}-1} \end{array}\right]}^{T}\cdot \Delta u_{k}.$$

Substituting Equation (22) into (20) yields

(23) $$\begin{array}{c} \Delta u_{k}={\left({L^{\prime }}^{T}S_{p}L^{\prime }+{\alpha_{s}}^{T}S_{q}\alpha^{\prime }\right)}^{-1}{L^{\prime }}^{T}S_{p}E\\ E=y_{\mathrm{rd}}-G\Delta z_{k}-C{\tilde{z}}_{k},\Delta z_{k}=z_{k}-z_{k-1} \end{array},$$

which subjects to

$${\rho^{\prime }}_{1}\cdot \Delta u_{k}\leq \rho_{2},z_{k-1}={\left[x_{k-1}^{T},{\left(\Lambda ({ϒ}_{k-1},\ldots ,{ϒ}_{k-m})\right)}^{T}\right]}^{T},z_{k}={\left[x_{k}^{T},{\left(\Lambda ({ϒ}_{k},\ldots ,{ϒ}_{k-m+1})\right)}^{T}\right]}^{T}.$$

The definitions of parameters in the controller are detailed in Appendix A. Considering the presence of unmeasurable disturbances, the model of system is extended to

(24) $$\left\{\begin{array}{l} z_{k}=M_{K}z_{k-1}+N_{K}u_{k-1}+w_{k}\\ y_{k}=C_{K}z_{k}+o_{k} \end{array}\right.,$$

where ***w*** and ***o*** represent the prediction noise and measurement noise, respectively. The output vector $y={\left[v,i_{A},i_{B}\right]}^{T}$. And the output coefficient matrix

(25) $$C_{K}=\left[\begin{array}{lllll} 1 & 0 & 0 & \ldots & 0\\ 0 & 1 & 0 & \ldots & 0\\ 0 & 0 & 1 & \ldots & 0 \end{array}\right],$$

**Figure 4:**
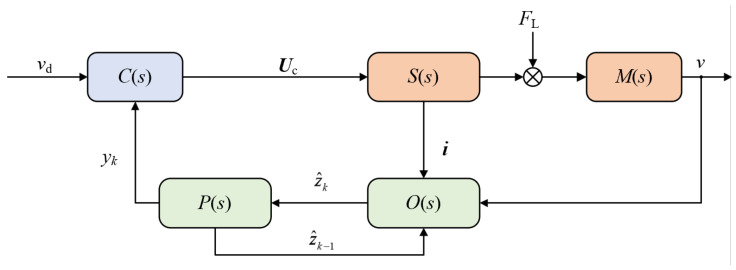
Block diagram of AKOBMPC algorithm.

The block diagram of the AKOBMPC algorithm is illustrated in Figure 4. *v*_d_ represents the given velocity trajectory, and *C*(*s*) denotes the MPC controller, which outputs the voltage amplitude *U*_c_. *P*(*s*) is the GLSP model with the output *y*_k_. *O*(*s*) denotes the function of AKOB, ${\hat{z}}_{k-1}$ and ${\hat{z}}_{k}$ represent the state estimation vector from the previous time step and the state vector after feedback correction, respectively. *S*(*s*) and *M*(*s*) represent the models of the stator and the mover.

### Key Assumptions, Application Scope and Limitations

1. Core Assumptions and Experimentally Validated Working Range

This work is established on four core theoretical assumptions: the core global linearization assumption holds that the inherent nonlinear dynamics of PEA can be embedded into a linear invariant subspace, with this framework validated under excitation voltages of 0–5 V and excitation frequencies of 0.5–5 Hz; the dead zone mapping assumption posits that the velocity dead zone of the PEA presents a consistent, asymmetric piecewise nonlinear relationship, with the proposed model identified and tested under external loads of 0–2.0 N; for the friction contact model, it is assumed that the contact interface between the stator and mover maintains a stable micro stick-slip friction state without macroscopic sliding under the tested loads of 0–2.0 N; finally, the disturbance estimation assumption approximates unmeasurable load disturbances and measurement noise as slow time-varying, zero-mean white noise processes, where the system operates at a fixed sampling frequency of 2.50 kHz, and the adopted GLSP model incorporates a historical state sequence with a window length set as a training hyperparameter to adequately capture the system dynamics.

2. Inapplicable Scenarios and Potential Failure Analysis

The proposed method may experience significant performance degradation or functional failure under the following typical operating conditions: high-frequency excitation that induces high-order modal coupling to invalidate the global linearization assumption, leading to a sharp increase in GLSP prediction error and potential closed-loop instability; excessive load that causes overall slip at the contact interface to violate the dead zone mapping and friction assumptions, resulting in elevated steady-state tracking error and even actuator stall; significant temperature drift that alters piezoelectric material parameters and friction coefficients to shift the dead zone threshold, leading to notable steady-state errors and reduced prediction accuracy; and abrupt friction state changes caused by severe wear, lubrication failure, or sudden preload variations, which completely modify the system dynamics and result in model prediction failure and system oscillation.

3. Parameter Re-identification and Update Rules

A hierarchical parameter re-identification and update rule is established to maintain the stable control performance of the proposed framework: complete offline re-identification of GLSP model weights and Koopman matrices is required when the PEA is mounted on a new platform or the friction interface undergoes significant changes; adaptive online real-time update of the noise covariance matrices is implemented via the AKOB for working condition variations within the effective range; and only semi-online fine-tuning of the dead zone voltage threshold is needed for gradual environmental changes such as slow temperature drift, with all core model parameters remaining fixed.

## 4. Result and Discussion

### 4.1. Experiment Setup

**Figure 5:**
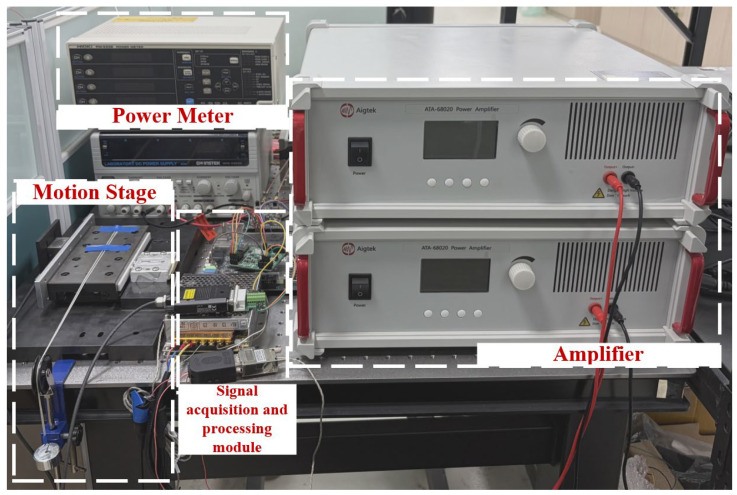
Experimental equipment.

The experimental platform is illustrated in Figure 5. The dual-mode standing wave PEA is a commercial actuator (Nanomotion Ltd., Yokneam Illit, Israel, Nanomotion, SE4 series), and the piezoelectric material is self-made by the company. First, the PEA and the mover (cross roller guide rail) were bolted onto a custom-designed aluminum alloy platform. Then, this integrated platform was rigidly mounted onto a high-precision optical isolation table to minimize environmental vibrations. A variable load force (*F*_L_) is applied to the stage via a fixed pulley using standard weights. The host computer is employed to implement the control algorithms and send instructions to the FPGA control board, with a sampling rate of 2.50 kHz. The control signals are amplified by the power amplifiers (Xi’an Aigtek Electronics Technology Co., Ltd., Xi’an, China, ATA-68020) to drive the actuator and actuate the mover. The displacement signal of the mover is captured by a displacement encoder (Dr. Johannes Heidenhain GmbH, Traunreut, Germany, LIP201) and transmitted back to the control board through the position manager board (Texas Instruments, Dallas, TX, USA, TIDM-1008). Additionally, driving current and voltage signals are monitored through an acquisition circuit with resolutions of 0.1 mA and 1 mV, respectively. Finally, the control board transmits the collected dataset information to the upper computer.

### 4.2. Identification and Verification of the Prediction Model

**Figure 6:**
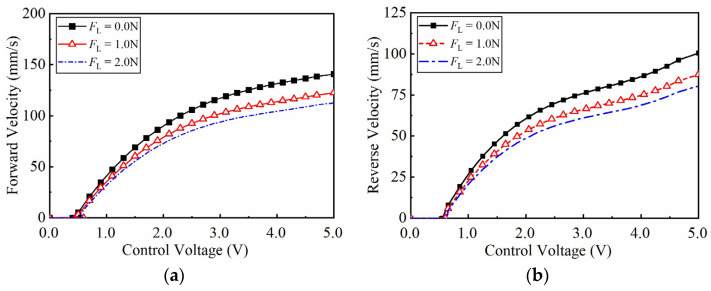
Open-loop velocity–voltage characteristics of the PEA system under constant voltages and varying loads. (**a**) Forward motion velocity curves; (**b**) reverse motion velocity curves.

**Figure 7:**
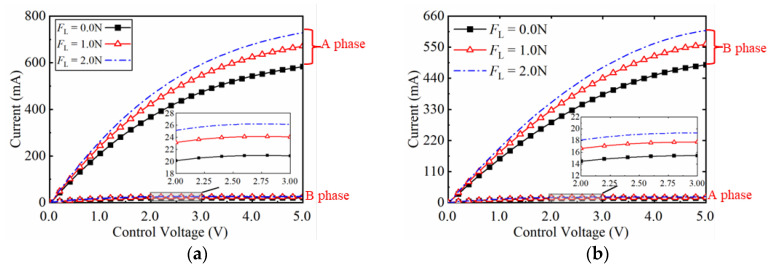
Open-loop two-phase current-voltage characteristics of the PEA system under constant voltages and varying loads. (**a**) A-phase and B-phase current curves during forward motion; (**b**) A-phase and B-phase current curves during reverse motion.

The experiment dataset is collected to identify the parameters of the GLSP model. The control voltage *u*_Ac_ or *u*_Bc_ is amplified by a power amplifier to excite the system to generate forward or reverse motion. As the constant control voltage is applied, Figure 6 shows the forward and reverse velocity curves under varying loads. The results clearly exhibit a dead zone characteristic. Furthermore, the two-phase currents are collected under open-loop condition. As shown in the steady-state current curves in Figure 7, the A-phase current is significantly higher than B-phase during forward motion, while the opposite trend is observed during reverse motion.

**Figure 8:**
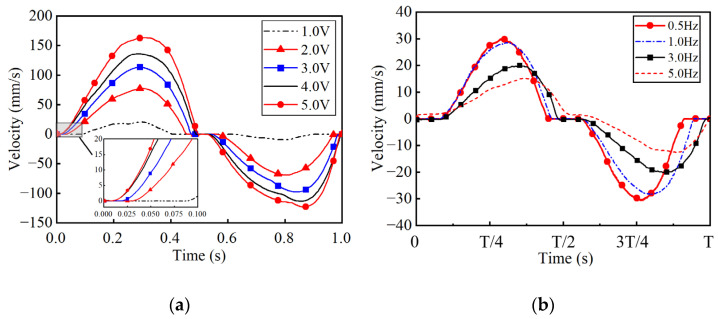
Open-loop dynamic velocity response of the PEA system under sinusoidal envelope voltages (no-load condition). (**a**) Velocity responses with voltage amplitudes 1–5 V at a fixed frequency of 1 Hz; (**b**) Velocity responses with frequencies 0.5–5 Hz at a fixed amplitude of 1 V.

As the sinusoidal envelope control signal *u*_c_ =*U*_c_ sin(2π*f*_c_*t*) was applied, where *U*_c_ and *f*_c_ stand for the excitation amplitude and frequency. Figure 8a shows the output characteristics no-load conditions with amplitudes ranging from 1–5 V (*f*_c_ = 1 Hz). The output characteristics for varying frequencies at *U*_c_ = 1 V are shown in Figure 8b. The resulting data indicates that the dead zone and nonlinear characteristics fluctuate significantly with changes in signal amplitude and frequency. The experimental data in Figure 6, Figure 7 and Figure 8 are used as the dataset for GLSP model identification.

The linearly normalized dataset with a total of 1.2 million sampling points is divided into training, validation, and test sets with a ratio of 6:2:2. The GLSP model is implemented through the TensorFlow framework with an Adam optimizer. The initial learning rate is set to 0.001, the first-order and second-order decay rates are 0.9 and 0.999, respectively, and the total training iterations are 900. The dimension of state observation is 64. The number of hidden layer neural nodes in the two-layer LSTM structure are 30 and 80, respectively. The weight coefficients *s*_1_ = *s*_2_ = *s*_3_ = 0.5.

The pruning block size is set to 4, which matches the four gate structures (input gate, forget gate, cell gate, output gate) of the LSTM unit, ensuring the integrity of the gate control logic after pruning. The iterative gradual pruning strategy is adopted with a total of five pruning iterations, and pruning is performed every 10 epochs during training, to avoid sudden performance degradation caused by one-time aggressive pruning. According to Algorithm 1, multi-step iterative pruning is performed on the neural network. The initial pruning rate *S*_0_ = 0, and the final target sparsity *S*_f_ =0.8, the total number of pruning steps *n* = 8, the pruning frequency Δ*t* = 50 epochs, and the initial training steps *t*_0_ = 200 epochs. During the training process, the pruning rate is gradually increased according to Equation (18) until the target sparsity is achieved.

The RMSVME is defined as

(26) $$\mathrm{RMSVME}=\sqrt{\frac{1}{N}\sum_{i=1}^{N}{\left(v_{m}\left(i\right)-v_{p}\left(i\right)\right)}^{2}},$$

where *v*_m_ and *v*_p_ denote the measured velocity and predicted velocity, respectively. *N* represents the number of data samples.

**Figure 9:**
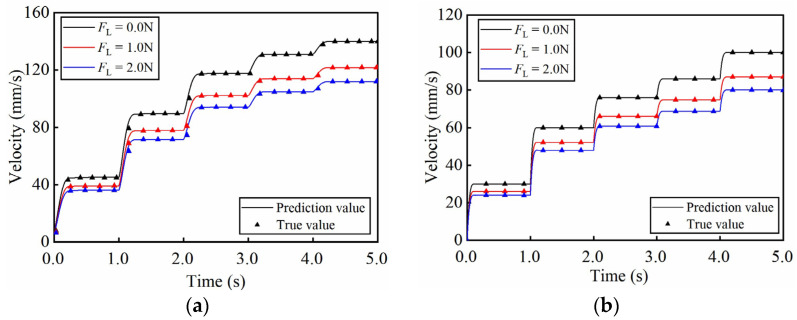
Prediction results under constant control voltage (**a**) The forward velocity curves; (**b**) The reverse velocity curves.

The prediction results under constant control voltage are illustrated in Figure 9. And Table 2 lists the RMSVMEs of the results. The model demonstrates high accuracy in reproducing forward and reverse motion under various loads. Specifically, the RMSVME remains below 0.32 mm/s for all tested loads, indicating robust predictive capability.

**Figure 10:**
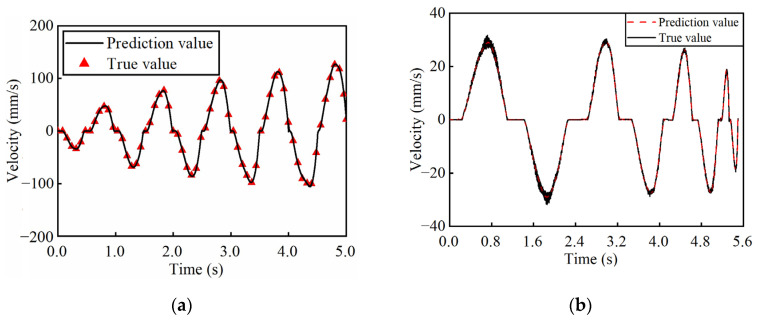
Prediction results under sinusoidal envelop control voltages (**a**) at varying amplitudes; (**b**) at varying frequencies.

In addition, the model is validated under sinusoidal envelop control voltage to assess the performance with a load of 2.0 N as shown in Figure 10. In the case of varying amplitude, under a constant frequency of 1 Hz, the RMSVME is 0.59 mm/s within the tested amplitude range. The RMSVME is 0.52 mm/s under sinusoidal envelope control voltages at varying frequencies.

Based on the experimental data, the proposed GLSP model maintains the RMSVME below 0.32 mm/s under loads from 0 to 2.0 N. Furthermore, the model achieves RMSVME of 0.59 mm/s under varying voltage amplitudes and 0.52 mm/s under varying excitation frequencies. The low prediction error under different loads and variable working conditions confirms its good generalized performance and working condition adaptability.

### 4.3. Design and Verification of Control Algorithm

In Algorithm 2, the initial values are set as $\hat{z_{0}}\;$= **0**_64×_, ***u***_0_ = **0**_2×1_, *ζ*= 0.95, ***P***_0_ = 10^−3^⋅***I***_64×64_, ***Q***_0_ = 10^−4^⋅***I***_64×64_, ***R***_0_ = 10^−3^⋅***I***_3×3_, ***λ***_0_ = 1, ***S***_p_ = ***I***_180×180_, ***S***_q_ = 0.0125⋅***I***_20×20_. The horizons are *M* = 60 and *M*_u_ = 10, which are the optimal values obtained through systematic simulation scanning optimization. The parameters for the controller are selected as α_s_ = 0.6, ζ=0.95. The constraints *b*_xu_ = 200, *b*_xl_ = −200, $b_{\mathrm{ul}}^{\prime }\;=\;{\left[0,\;0\right]}^{T}$, $b_{\mathrm{uu}}^{\prime }\;=\;{\left[5,\;5\right]}^{T}$, Δ*b*_ul_ = [0, 0]^T^, Δ*b*_uu_ = [0.5, 0.5]^T^.

To evaluate the steady-state tracking performance, the SRMSVE is defined as

(27) $$\mathrm{SRMSVE}=\sqrt{\frac{1}{N_{s}}\sum_{k=1}^{N_{s}}{\left(v_{\mathrm{rd}}\left(k\right)-v_{ac}\left(k\right)\right)}^{2}},$$

where *v*_rd_ and *v*_ac_ denote the reference velocity and actual output velocity, respectively. *N*_s_ represents the number of data samples.

**Figure 11:**
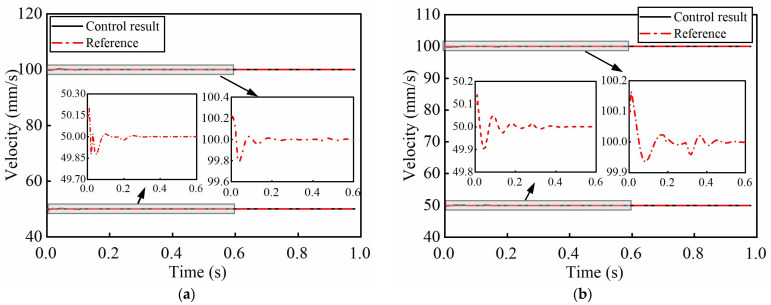
Closed-loop control simulation results under constant velocity references. (**a**) The forward velocity curves; (**b**) the reverse velocity curves.

Figure 11 shows the simulation results of the control scheme under the constant reference velocity. For the forward motion, when the reference velocity amplitude are 50.00 mm/s and 100.00 mm/s, the maximum velocity errors are 0.20 mm/s and 0.23 mm/s, and the SRMSVE are 0.03 mm/s and 0.04 mm/s, respectively. For the case of reverse motion, when the amplitudes are 50.00 mm/s and 100.00 mm/s, the maximum velocity error values are 0.14 mm/s and 0.18 mm/s, and the SRMSVE are 0.02 mm/s and 0.03 mm/s, respectively. These preliminary findings indicate that the proposed controller can effectively track the reference trajectory, and significantly reduce the nonlinear characteristics of the system.

**Figure 12:**
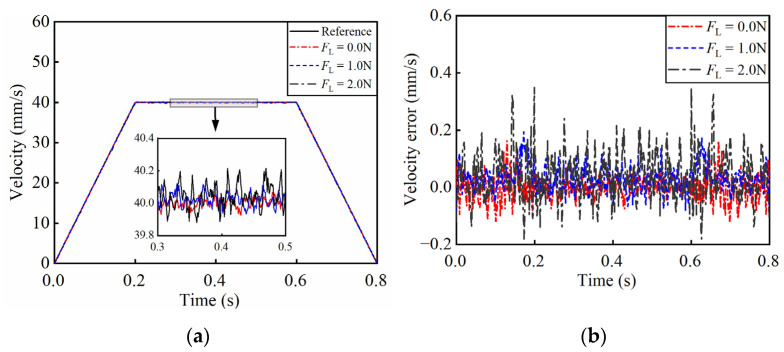
Closed-loop control results under trapezoidal references. (**a**) The velocity curves; (**b**) the velocity error curves.

**Figure 13:**
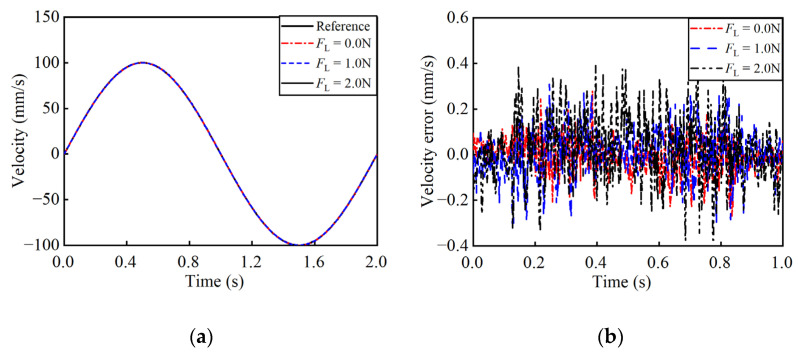
Closed-loop control results of sinusoidal reference velocity trajectory. (**a**) The velocity curves; (**b**) the velocity error curves.

To verify the performance of the algorithm, the experimental validations are conducted under varying loads and reference trajectories. Figure 12 shows the control results of the trapezoidal reference velocity trajectory. When the reference velocity is 40.00 mm/s, under loads of 0 N, 1.0 N, and 2.0 N, the actual velocity fluctuates within a narrow range of 39.65–40.35 mm/s. And the SRMSVE are 0.04 mm/s, 0.05 mm/s and 0.07 mm/s, respectively. Figure 13 depicts the tracking performance for a sinusoidal reference. At a peak velocity of 100.00 mm/s, the actual velocity remains within the range of 99.50–100.50 mm/s. And the SRMSVE are 0.08 mm/s, 0.10 mm/s, and 0.11 mm/s, respectively.

At a reference velocity of 100.00 mm/s, the proposed scheme is compared with existing control algorithms in [37,38], and the results are shown in Table 3. It can be seen that the proposed AKOBMPC algorithm can accurately track the reference velocity trajectory and effectively solve the problem of output nonlinearity in the system, which is of crucial significance for high-precision displacement tracking applications.

Compared with the existing control methods, the proposed AKOBMPC algorithm reduces the maximum tracking error by more than 44%. And the SRMSVE is suppressed to 0.11 mm/s. The excellent steady-state performance mainly benefits from two aspects: (1) the GLSP model accurately predicts the dead zone nonlinearity of the PEA system, which eliminates the steady-state error caused by the unmodeled dead zone characteristics; (2) the AKOB estimates and compensates the unmeasurable load disturbance and measurement noise in real time, which ensures the stability of the state feedback and further reduces the steady-state fluctuation of the system.

The excellent tracking performance and disturbance rejection capability of the proposed scheme stem from the synergistic optimization of three core modules: the Koopman-based global linearized framework enables high-fidelity modeling of the complex electromechanical coupling and dead zone nonlinear dynamics to lay a reliable model foundation for the MPC controller; the structured pruning strategy effectively balances the prediction accuracy and real-time computational efficiency of the GLSP model to ensure its feasibility for real-time embedded deployment; and the adaptive Kalman observer realizes real-time estimation and compensation of unmeasurable load disturbances and measurement noise, significantly enhancing the robustness and anti-disturbance performance of the closed-loop system.

## 5. Conclusions

In this study, a novel Koopman-based GLSP model and an AKOBMPC scheme are developed to address the inherent dead zone nonlinearities and time-varying disturbances in a PEA system. The primary conclusions are as follows:

(1) The proposed GLSP model successfully maps the complex, nonlinear electromechanical coupling and contact dynamics of the PEA into a linear invariant subspace. By employing a structured pruning algorithm, the model maintains high prediction accuracy while achieving computational efficiency suitable for real-time control.
(2) Integration of the AKOB provides real-time estimation and compensation for unmeasurable disturbances and measurement noise. This enhances the reliability of state feedback, even under varying load conditions from 0 N to 2.0 N.
(3) Experimental validation demonstrates that the AKOBMPC algorithm effectively eliminates the velocity dead zone. For a trapezoidal reference, fluctuations are suppressed within ±0.35 mm/s. Under a sinusoidal reference with a peak velocity of 100.00 mm/s, the maximum tracking error is reduced to 0.50 mm/s.

Overall, the proposed framework offers a robust and high-precision solution for the motion control of a PEA, demonstrating strong potential for applications that demand high positioning and stable velocity regulation in dynamic environments. The control architecture also exhibits good scalability, which supports its future extension to the high-precision motion control of piezoelectric robots.

## Figures and Tables

**Table 1:** The computational complexity of each part in the network.

Definition	Parameter Quantity
Input cache	(*h*_1_ + *h*_2_)
Weight matrix cache	4·((*n* + *h*_1_)·*h*_1_ + *h*_1_ + (*h*_1_ + *h*_2_)·*h*_2_ + *h*_2_)
Bias vector cache	4·(*h*_1_ + *h*_2_)
Cell state cache	(*h*_1_ + *h*_2_)
Output cache	(*h*_1_ + *h*_2_)
Total cache	4·((*n* + *h*_1_)·*h*_1_+ (*h*_1_ + *h*_2_)·*h*_2_)+ 8·(*h*_1_ + *h*_2_)

**Table 2:** The RMSVME of the velocity curves under the constant control voltage.

Load (N)	Forward RMSVME (mm/s)	Reverse RMSVME (mm/s)
0.0	0.23	0.22
1.0	0.31	0.32
2.0	0.25	0.24

**Table 3:** Comparison of velocity tracking performance.

Control Algorithm	Maximum Velocity Error (mm/s)
[37]	1.17
[38]	0.90
The proposed controller	0.50

## Data Availability

Data available on request from the authors.

## Abbreviations

The following abbreviations are used in this manuscript:

AKOB	Adaptive Kalman Observer
AKOBMPC	Adaptive Kalman Observer-based Model Predictive Control
DEP	Dimension Enhancement Part
DRP	Dimension Restoration Part
GLSP	Global Linearized Sparse Prediction
LEP	Linear Evolution Part
LSTM	Long Short-Term Memory
MPC	Model Predictive Control
PEA	Piezoelectric Actuator
PI	Prandtl–Ishlinskii
RMSVME	Root Mean Square Velocity Modeling Error
SRMSVE	Steady-State Root Mean Square Velocity Error

## Appendix A. Parameter Matrices for the AKOBMPC Scheme

(A1)
$$\rho_{1}^{\prime }=\rho_{1}\cdot \alpha_{s},$$

(A2)
$$G=\left[\begin{array}{c} C_{K}M_{K}\\ C_{K}M_{K}^{2}+C_{K}M_{K}\\ \vdots \\ \sum_{i=1}^{M}C_{K}M_{K}^{i} \end{array}\right],C=\left[\begin{array}{cccc} C_{K} & 0 & \ldots & 0\\ 0 & C_{K} & \ldots & 0\\ \vdots & \vdots & \ddots & \vdots \\ 0 & 0 & \ldots & C_{K} \end{array}\right],$$

(A3)
$$\rho_{1}=\left[\begin{array}{c} I_{M_{u}\times M_{u}}\\ -I_{M_{u}\times M_{u}}\\ P_{2}\\ -P_{2}\\ L\\ -L \end{array}\right],\rho_{2}=\left[\begin{array}{c} \Delta b_{uu}\\ -\Delta b_{ul}\\ b_{uu}-P_{1}u_{k-1}\\ -b_{ul}+P_{1}u_{k-1}\\ b_{xu}-G\Delta z_{k}-Cz_{k}\\ -b_{xl}+G\Delta z_{k}+Cz_{k} \end{array}\right],P_{1}=\left[\begin{array}{c} I\\ I\\ \vdots \\ I \end{array}\right],P_{2}=\left[\begin{array}{cccc} I & 0 & \ldots & 0\\ I & I & \ldots & 0\\ \vdots & \vdots & \ddots & \vdots \\ I & I & \ldots & I \end{array}\right],$$

(A4)
$$L=\left[\begin{array}{cccc} C_{K}N_{K} & 0_{H\times 1} & \ldots & 0_{H\times 1}\\ C_{K}M_{K}N_{K}+C_{K}N_{K} & C_{K}N_{K} & \ldots & 0_{H\times 1}\\ \vdots & \vdots & \ddots & \vdots \\ \sum_{i=0}^{M-1}C_{K}M_{K}^{i}N_{K} & \sum_{i=0}^{M-2}C_{K}M_{K}^{i}N_{K} & \ldots & \sum_{i=0}^{M-M_{u}}C_{K}M_{K}^{i}N_{K} \end{array}\right]$$
